# Dental implant surface temperatures following double wavelength (2780/940 nm) laser irradiation in vitro

**DOI:** 10.1002/cre2.369

**Published:** 2020-12-04

**Authors:** Peter Fahlstedt, Dagmar F Bunæs, Stein Atle Lie, Knut N Leknes

**Affiliations:** ^1^ Faculty of Medicine, Department of Clinical Dentistry University of Bergen Bergen Norway; ^2^ Faculty of Medicine, Department of Clinical Dentistry –Periodontics University of Bergen Bergen Norway

**Keywords:** calibration, dental implant, laser, temperature, titanium

## Abstract

**Objective:**

To estimate the implant surface temperature at titanium dental implants during calibrated irradiation using double wavelength laser.

**Material and methods:**

A double wavelength laser, 2780 nm Er,Cr:YSGG and 940 nm diode, was calibrated and used to irradiate pristine titanium dental implants, OsseoSpeed, TiUnite and Roxolid SLActive, representing different surface modifications. Initial calibration (21 implants; 7 implants/group) intended to identify optimal wavelength/specific output power/energy that not critically increased the temperature or altered the micro‐texture of the implant surface. Subsequent experimental study (30 implants; 10 implants/group) evaluated implant surface temperature changes over 190 s. Irradiation using a computerized robotic setup.

**Results:**

Based on the initial calibration, the following output powers/energies were employed: Er,Cr:YSGG laser 18.4 mJ/pulse (7.3 J/cm^2^)–36.2 mJ/pulse (14.4 J/cm^2^) depending on implant surface; diode laser 3.3 W (1321.0 W/cm^2^). During double wavelength irradiation, implant surface temperatures dropped over the first 20 s from baseline 37°C to mean temperatures ranging between 25.7 and 26.3°C. Differences in mean temperatures between OsseoSpeed and TiUnite implants were statistically significant (*p* < 0.001). After the initial 20 s, mean temperatures continued to decrease for all implant surfaces. The decrease was significantly greater for TiUnite and Roxolid SLActive compared with OsseoSpeed implants (*p* < 0.001).

**Conclusion:**

Calibrated double wavelength laser irradiation did not critically influence the implant surface temperature. During laser irradiation the temperature decreased rapidly to steady‐state levels, close to the water/air‐spray temperature.

## 1 INTRODUCTION

Titanium surface decontamination using various laser systems have been advocated (Kamel et al., 2014). Thus Er:YAG (2940 nm) and Er,Cr:YSGG (2780 nm) lasers have been deemed suitable for biofilm and calculus removal producing minimal, if any, surface alterations (Taniguchi et al., 2013; Takagi et al., 2018; Larsen et al., 2017), decontamination directly proportional to applied energy (J) and fluence (J/cm^2^) (Ercan et al., 2014; Al‐Hashedi et al., 2017; Monzani et al. 2018). Moreover, diode lasers of different wavelengths have been shown to yield high antimicrobial effects following irradiation of cultured biofilm on titanium surfaces without modifying the surface topography (Bach et al., 2000; Deppe & Horch, 2007).

However, irradiation of titanium dental implants using laser energy may lead to unintended heating of the implant potentially compromising osseointegration. Animal studies suggest a 47°C exposure over 1 min a critical threshold to inflict damage to bone (Eriksson & Albrektsson, 1983, 1984). Only a few studies examining Er,Cr:YSGG lasers and none the 940 nm diode laser or double wavelength laser have been presented relative to their thermal effects on titanium dental implants (Gomez‐Santos et al., 2010; Romanos et al., 2017) or titanium discs (Ercan et al., 2014; Strever et al., 2017). By contrast, in several studies, thermal effects on different titanium surfaces have been evaluated during or after irradiation of pulsed 2940 nm Er:YAG laser (Monzani et al. 2018; Hakki et al., 2017) and diode lasers of 810, 980 and 1064 nm (Geminiani et al., 2012; Leja et al., 2013; Matys et al., 2016; Valente et al., 2017). These studies suggest that by using water‐spray cooling, the implant temperature may not exceed 47°C. As each wavelength has a unique curve for the absorption coefficient in different tissues and substances, a comparison between the properties of two neighboring lasers in the electromagnetic spectrum is only approximate (Valente et al., 2017). Moreover, a study on titanium dental implants placed in porcine bone concluded that thermal conductivity as well depends on the chemical composition and diameter of the implant (Matys et al., 2016).

Literature reviews fail to consent on preferred protocols for safe laser debridement and disinfection of contaminated dental implants. Heterogeneity in protocol, variation in included parameters, and lack of information concerning calibration of the laser equipment and measuring instruments hamper comparisons between studies (Kamel et al., 2014; Smeo et al., 2018). Most laser systems report a discrepancy between device power/energy setting and actual output power/energy. Variables including laser light transmission system, output power/energy, pulse rate, laser beam area and divergence (spread), and distance to irradiated surface need to be identified and optimized prior to clinical application (Takagi et al., 2018; Tunér & Jenkins, 2016).

The effect of double wavelength laser irradiation has to our knowledge not been reported for titanium dental implants. Acknowledging the lack of laser specific information, the overall aim of this study was to investigate whether double wavelength laser irradiation critically increases the implant surface temperature above the critical threshold of 47°C for different implant systems using a validated in vitro protocol. We hypothesized that surface temperature would rapidly increase, but not above the critical threshold of 47°C. The null hypothesis was that the final temperature would be similar for the different implant systems. This study consists of two parts: First, to identify the output handpiece fiber tip power and energy for each implant system that do not produce thermal heating or surface micro‐texture alterations, and second, to evaluate implant surface temperature for principal implant systems using calibrated irradiation.

## 2 MATERIAL AND METHODS

### 2.1 Titanium dental implants

Titanium dental implants, representing three principal implant systems featuring different surface characteristics, were evaluated in vitro. The sample size for the experimental part of the study, was based on findings obtained from the calibration part. To account for unexpected implant variation, we aimed at *n* = 10.

**FIGURE 1:**
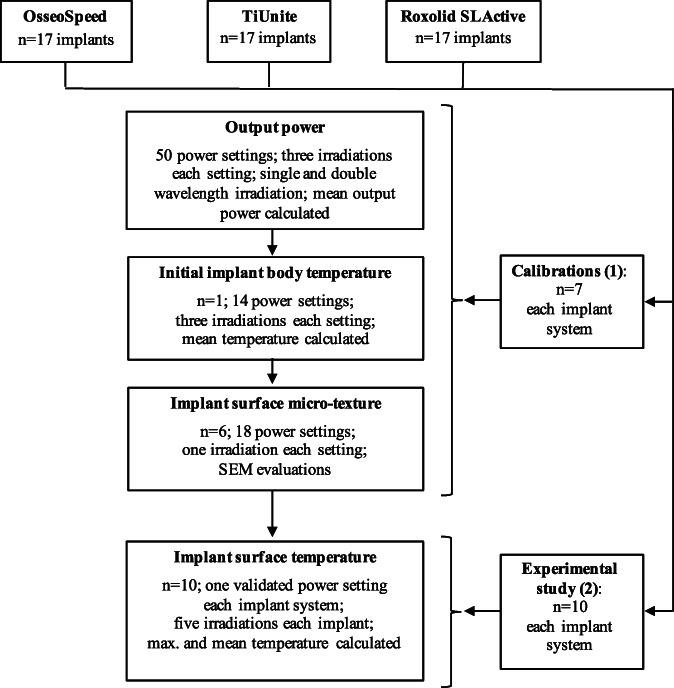
Flow chart of study. Part 1: Calibrations of the double wavelength laser irradiation; Part 2: Experimental study using double wavelength laser irradiation

Twenty‐one implants, seven for each system, were used for calibration. Thirty implants, 10 for each system, were used for the experimental study (Figure 1). All implants were received sterile in the manufacturers original packaging: Nobel Biocare, TiUnite dental implants (D, 4.0 mm; L 13.0 mm; Replace Select TC, RP; Nobel Biocare AG, Kloten, Switzerland); Straumann SLA dental implants (D, 4.0 mm; L 12.0 mm; Roxolid SLActive, BL, Straumann AG, Basel, Switzerland); and Astra Tech dental implants (D, 4.0 mm; L 13.0 mm; OsseoSpeed TX; Dentsply Sirona Implants, AG, Salzburg, Switzerland).

### 2.2 Laser system

This study used a double laser that combines two wavelengths of laser light; one free running pulsed 2780 nm Er,Cr:YSGG laser and one 940 nm diode laser operating in continuous wave mode (Table 1; Biolase Inc., Irvine, CA).

**TABLE 1:** Properties of the lasers (manufacturer's information)

Laser properties	Laser 1	Laser 2
Laser wavelength (nm)	2780	940 ± 10
Mode of operation	Free running pulsed	Continuous wave (CW)
Number of emitters	1	1
Emitter type	Er;Cr:YSGG	InGaAsP, semi‐conductor, diode
Power accuracy	± 20%	± 20%

Laser beam delivery system	
Beam delivery	Optical fibre
Beam profile	Gaussian beam
Handpiece	*Gold*, for fibre tips
Fibre tip	*MZ8*, length 6 mm, diameter 800 μm
Divergence angle fibre tip	8°
Numerical aperture, fibre tip	0.14

The following settings were used during calibration and the experimental study: Er,Cr:YSGG–pulse duration 60 μs, repetition rate 50 Hz (Al‐Karadaghi et al., 2015; Gutknecht et al., 2016); 940 nm diode laser–continuous wave mode (CW). When water/air spray cooling was used, the device setting was 80% water–20% air.

### 2.3 Calibration

#### 2.3.1 Output power

A calibrated thermal sensor (FL250A‐BB‐50, Ophir Photonics, Darmstadt, Germany) and universal power meter (Vega Standard, P/N 7Z01560, Ophir Photonics) were used to measure the output power from the laser handpiece fiber tip at a 1‐mm distance without water/air spray. With one laser wavelength (single) activated at each time, output power was measured for the pulsed Er,Cr:YSGG laser and for the 940 nm diode laser (CW) using 10 different power settings (1.0–4.25 W). Double wavelength irradiation was measured at 10 power settings for Er,Cr:YSGG laser from 1.0 to 4.25 W in combination with three diode laser settings (1.0 W; 2.25 W; 4.25 W) and compared to the same settings as single wavelength irradiation. In total, 50 different power settings repeated thrice were tested.

#### 2.3.2 Initial implant body temperature

One implant from each system was used for body temperature measurements following irradiation with Er,Cr:YSGG and 940 nm diode laser as single and double wavelength laser. An oscillating movement in a half turn pattern at a speed of approximately 8.2 mm/s over an area of 24 mm^2^ was used to approximate clinical settings. The implants, attached to a hollow pin, were driven by an endodontic handpiece connected to an electric motor. One thermo‐coupler was fixed in the center of the implant and the implant temperature adjusted to 37°C using a fan. Final temperature following a 30‐s irradiation was displayed on a thermometer logger.

Three power settings for each laser wavelength (1.0 W; 2.25 W; 4.25 W) were tested at the fixed distance of 1.0 (±0.2) mm. The two wavelengths were tested as single and double. Water/air‐spray was activated at all Er,Cr:YSGG irradiations. Diode laser irradiations were tested with and without water/air‐spray. In total, for each implant, 18 settings repeated thrice were performed for the implant body temperature measurements.

#### 2.3.3 Implant surface micro‐texture

A scanning electron microscope (SEM, Jeol JSM‐7400F, Tokyo, Japan) was used to evaluate surface micro‐texture alterations following irradiation with four (0.75 W–3.0 W) and two (1.00 W; 4.25 W) different power settings for the Er,Cr:YSGG and the 940 nm diode laser, respectively. Areas of non‐irradiated implant were used as control. The two lasers were tested for single and double irradiation.

Each implant was divided into four areas (Takagi et al., 2018). The 1.0‐mm distance between the fiber tip end and implant surface was controlled using a calibrated USB‐microscope (Dino‐Lite/Europe, Naarden, Netherlands) and output power controlled using the calibrated thermal sensor/power meter. A Computer Numerical Control (CNC) (Lase‐o‐Matic, Viking; ILSD Sweden AB, Stockholm, Sweden) prototype device was used for repeatable oscillation of the implants. The fixed laser handpiece in the CNC device had an angle of 90° relative to the implant surface, and a repeatable vertical movement pattern simulating clinical debridement. The speed of movement was calibrated to 3.3 mm/s, and each vertical step of every half turn was 0.5 mm. Irradiated area was 21 mm^2^ (3 × 7 mm, height × width). The setup was designed to ensure that each mm^2^ of implant surface was exposed to a minimum of 32 pulses from the Er,Cr:YSGG laser and 5 s of the continuous wave diode laser.

For the SEM evaluation, the implants were placed on sample studs and fully inspected at 5–10 kV at a magnification of ×50, ×190, and ×1500. Representative surface areas were recorded. Implant areas were then allocated into two groups based on the following surface descriptions: areas with no alteration (*No surface alteration group*) compared with control areas; areas with infractions, cracks, melting or ablations (*Surface alteration group*).

### 2.4 Experiment

The experimental study was conducted under validated settings based on the calibrations. All implants were irradiated at an angle of 90°, four turns in horizontal‐vertical direction on one side of the implants covering 42 mm^2^ (7 × 6 mm, height × width). The speed of movement was horizontally 3.3 mm/s, and each vertical step of every half turn was 0.5 mm.

**FIGURE 2:**
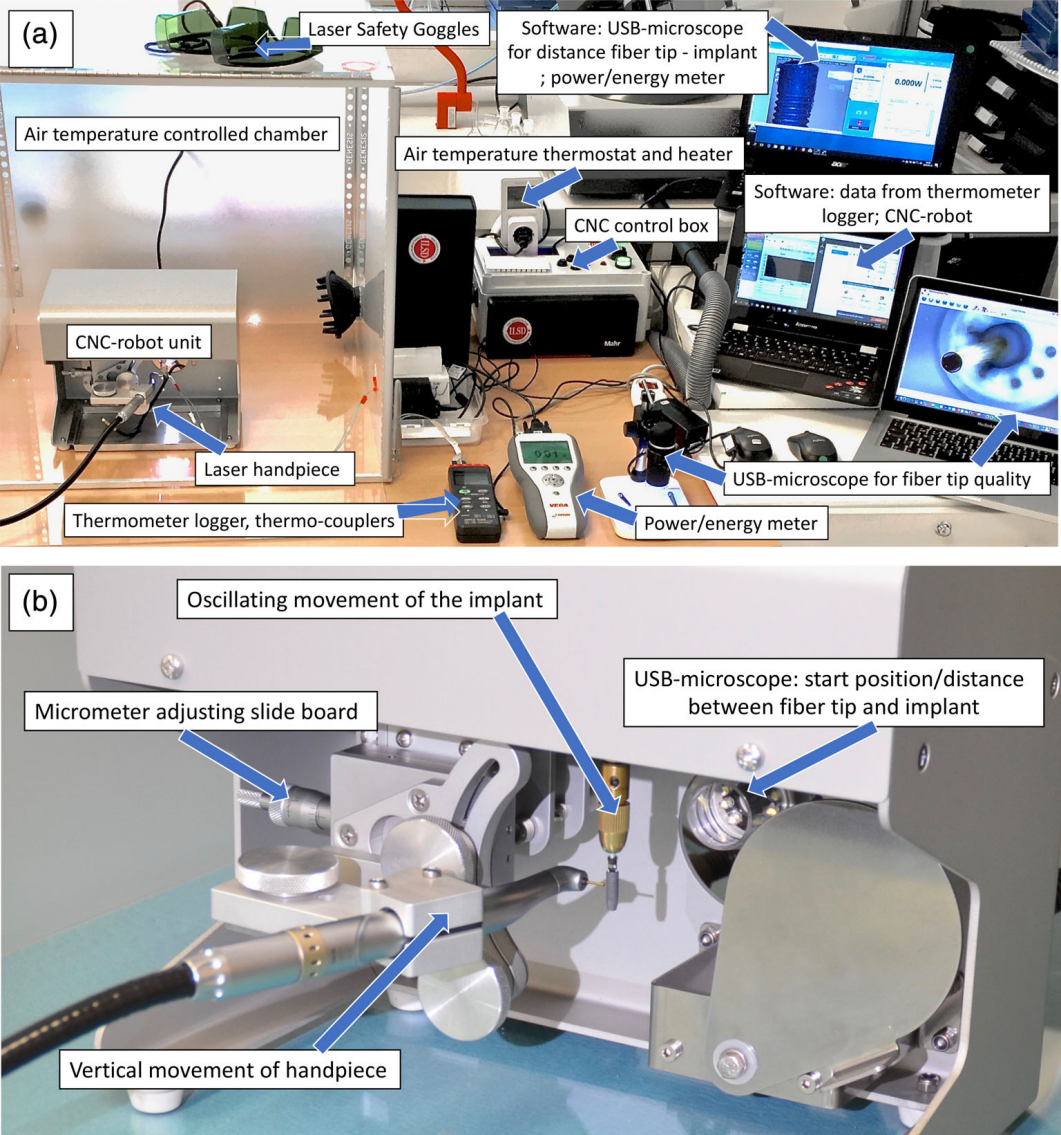
(a) Laboratory setup for the experimental study. (b) CNC‐robot unit

The start position of the fiber tip and the distance to the implant surfaces were adjusted, images recorded using the calibrated USB‐microscope fixed in the CNC‐device (Figure 2). Control of the fiber tip quality was recorded at ×200 using a USB microscope (Figure 2; DigiMicro Profi, DNT, Conrad Electronic International, Hirschau, Bavaria, Germany). Two calibrated thermo‐coupler K‐types (TC 309 S/N 151206276, SWEMA AB, Farsta, Sweden) with a ø0.5 mm contact tip and error rate of 0.5% were used to record the implant surface temperature.

A data logger thermometer (SWEMA AB, Farsta, Sweden) and a software interface (TestLink SE‐309‐USB, Norwalk, CT) had the ability to record the temperature at 1‐s intervals. The thermo‐couplers were attached by two layers of removable foam tape (3 M High‐Temperature Acrylic Adhesive, 4658f, 0.8 mm, Maplewood, TX) on the opposite side of the irradiated area and vertically positioned 2 mm above and below the midline of the implant. The dimension of the tape was adjusted to 8 × 6 mm (height × width) (Figure 2). A 600 × 600 mm acrylic chamber with a hatch in the front was used to control the thermal conditions.

Prior to irradiation, the temperature of the mounted implant, laser handpiece and CNC‐device were adjusted to 36.0–38.0°C using an air heater fan and thermostat in the chamber. At the time of irradiation, the temperature of the water in the tank for water/air‐spray and volume of applied water per min was recorded. Before and after each irradiation, output power from the fiber tip was recorded using the thermal sensor/power meter.

Each implant underwent five irradiations, each implant system irradiated 50 times (10 × 5). Activation of the double wavelength laser irradiation and recording of temperature started when the implant reached the baseline 36.0–38.0°C temperature and stopped after four completed turns of irradiated area. The air temperature in the chamber was controlled and maintained at 36.0–38.0°C throughout the test. A total irradiation period of 190 s was applied (Goncalves et al., 2010).

### 2.5 Statistical analysis

The methodology, results, statistical analysis and conclusions were reviewed, and all statistical analysis was performed blindly, by a professional statistician (SAL) who is also one of the study authors.

For calibration, mean output power for each power setting of double irradiation was compared with the sum of mean output power of single irradiation from the two wavelength lasers. The mean, standard error and *p*‐value of the differences were calculated. Intraclass correlations coefficients (ICCs), measuring the dependency between measures within each power setting, was calculated based on linear mixed effects models.

In the experiment, for each single implant, mean and max temperatures (measured after the first 20 s) were recorded. For comparison of max temperature between the implant systems, mixed effects models were applied. In this model, the implant system was entered as a fixed effect, while the implant number was entered as a random effect accounting for a possible dependency between measures. Post‐hoc analyses for comparisons of differences between treatment groups were adjusted using Scheffe's method. Results were considered statistically significant for *p* < 0.05. Based on the linear mixed effects models, ICCs measuring dependency within each individual implant was calculated. For the complete measures during the 190‐s interval, a mixed effects regression model was used to calculate mean effects and mean change (slope). The results from this model were presented graphically. For the maximum and mean temperature for each irradiation, mean, standard deviation and range were calculated, followed by a post‐hoc sample size calculation, based on the observed parameters using *t*‐tests. Data were analyzed using STATA version 15 (Stata Corp, Collage Station, TX).

## 3 RESULTS

### 3.1 Calibration

#### 3.1.1 Output power

Irrespective of power setting, estimated fiber tip output power was consistently lower than the device setting. The discrepancy ranged between 9.3% and 20.3% (mean = 17.3%) for Er,Cr:YSGG and between 13.0% and 23.8% (mean = 20.0%) for 940 nm diode laser. Mean differences in output power between double wavelength and the sum of two single wavelength irradiations for each power setting were small (mean = 0.02, SE = 0.39, *p* = 0.021). The ICC was high (0.99).

#### 3.1.2 Initial implant temperature

After 30 s irradiation with water/air‐spray, mean internal implant temperature ranged from 22.2 to 25.8°C below the critical 47°C temperature. Differences between the implant systems at same output power ranged from 1.5 to 1.7°C. The lowest temperature (21.2°C) was recorded for OsseoSpeed following single irradiation using the 940 nm diode laser at an output power of 0.9 W (setting 1.0 W), whereas the highest temperature (24.8°C) was recorded for TiUnite at 3.3 W (setting 4.25 W) following double wavelength irradiation.

For single diode laser irradiation without water/air‐spray, the temperature ranged between 6.4 and 105.4°C above 47°C. Differences in temperature between implant systems with the same output power ranged between 11.8 and 45.4°C. The lowest temperature was recorded for the diode laser irradiation with a 0.89 W output power (Roxolid SLActive 53.4°C, OsseoSpeed 63.2°C, and TiUnite 65.2°C), whereas the highest temperature was recorded with a 3.3 W output power (TiUnite 152.4°C, OsseoSpeed 150.0°C, and Roxolid SLActive 107.0°C).

#### 3.1.3 Implant surface micro‐texture

The SEM evaluation of the implants following diode laser irradiation did not reveal any alterations of the surface texture at any power setting; two surface areas for each implant system in *No surface alteration group* (output power 0.9 and 3.3 W).

For Er,Cr:YSGG laser irradiation, surface micro‐texture alterations appeared at different output powers pending implant system. In the *No surface alteration group*, there were one OsseoSpeed (1.1 W), one Roxolid SLActive (1.8 W) and two TiUnite areas (0.7 W and 0.9 W) (Table 2).

**TABLE 2:** Maximum laser settings and calculated power/energy based on calibrations not causing thermal heating or surface micro‐texture alterations

Laser/implant system	Device setting, power (W)	Output power^a^ (W)	Peak power^b^ (W)	Pulse energy (mJ/pulse)	Fluence/pulse^c^ (J/cm^2^)	Fluence/pulse^d^ (J/cm^2^)
Er,Cr:YSGG laser						
OsseoSpeed	1.3	1.1	360.0	21.6	8.6	4.3
TiUnite	1.0	0.9	306.7	18.4	7.3	3.7
Roxolid SLActive	2.3	1.8	603.3	36.2	14.4	7.2

					Intensity (W/cm^2^)	Intensity (W/cm^2^)
940 nm diode laser						
All implants	4.3	3.3	‐	‐	1321.0	660.5

*Note*: water/air‐spray volume: 29.2 ml/min.

^a^ Measured value.

^b^ Calculated values from output power.

^c^ Gaussian beam.

^d^ Flat beam (for compare).

**FIGURE 3:**
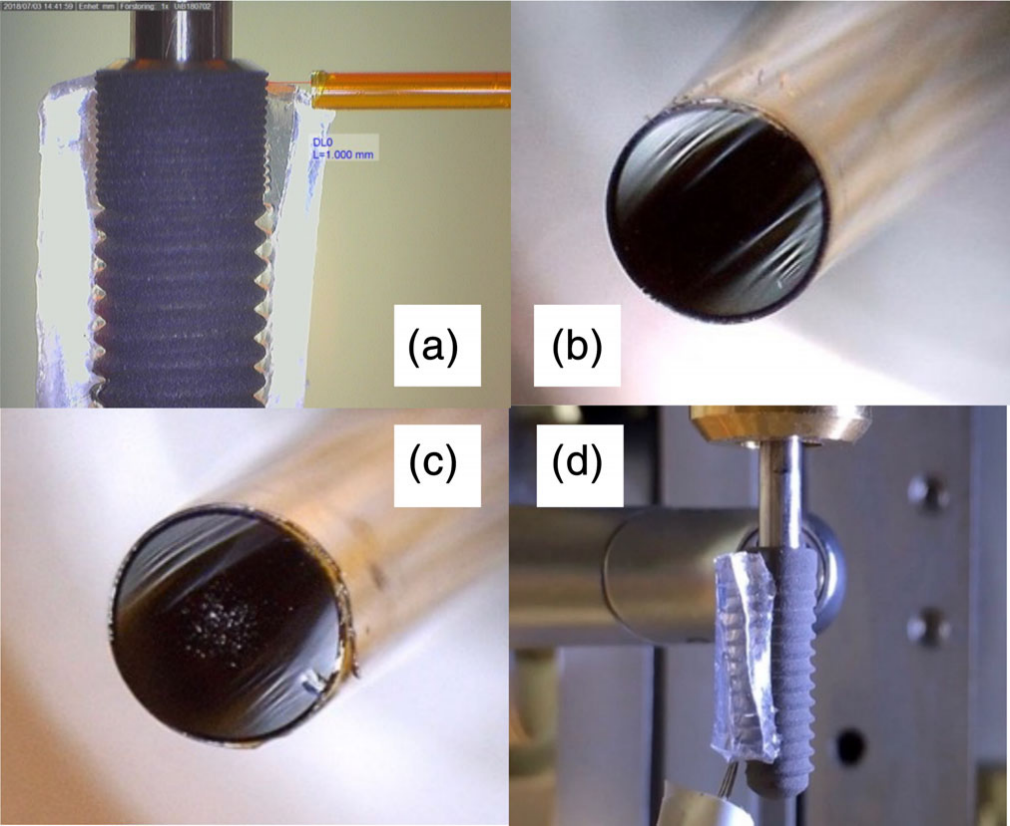
(a) Control and calibration of start position and distance between fibre tip end and outer line of implant at ×40. Laser handpiece fibre tip perpendicular to implant surface. (b) Quality control of the laser fibre tip at ×200. Diameter of the fibre tip 0.8 mm. New tip. (c) Used, slightly damaged tip. (d) Tight mounting of the thermal couplers by acrylic foam tape for optimal, durable adhesion and thermal isolation

In the *Surface alteration group*, there were three OsseoSpeed and Roxolid SLActive areas and two TiUnite areas. The area of altered surface increased, following an increase of output power (Figure 3). The first signs of alteration were minor and at different output powers, depending on implant system: OsseoSpeed (1.2 W) displayed melted TiO_2_ particles with rounded edges confined to the crests of the threads; TiUnite (1.1 W) displayed cracks and areas of total ablation of the TiO_2_ layer at the crest of the threads; Roxolid SLActive (2.0 W) disclosed droplet formed areas of Ti in the range of 2–10 μm on the prominent parts of the threads. At higher output power, more extensive alterations such as areas of melting TiO_2_ particles, totally ablated oxide layer and melted titanium droplets were observed.

### 3.2 Temperature experiment

**FIGURE 4:**
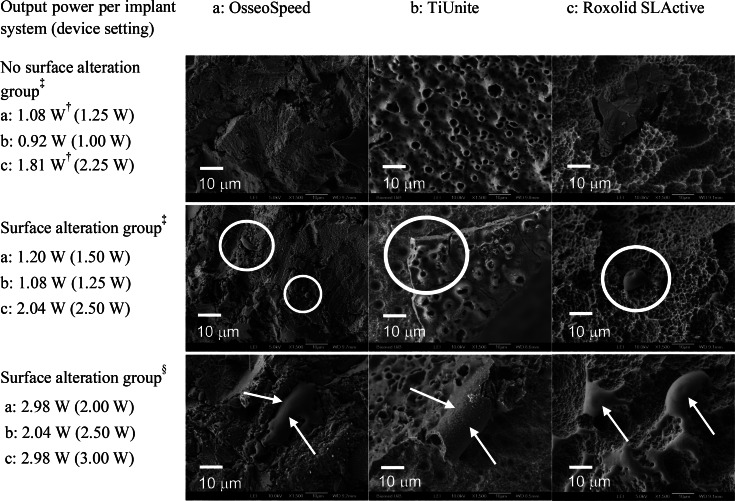
SEM images of surface micro‐texture alterations following single and double wavelength irradiation at different output powers of the Er,Cr:YSGG laser. Device setting in brackets. The 940 nm diode laser did not cause surface alterations at any output power. Minor observed signs of alteration (white circles); extensive alterations (white arrows). Magnification ×1500

For the experimental part, calibrated laser settings (Table 2) were used. From baseline temperature of 36.0–38.2°C, following 20 s of irradiation, the surface temperature for all implants dropped approximately 10°C (Figure 4).

**FIGURE 5:**
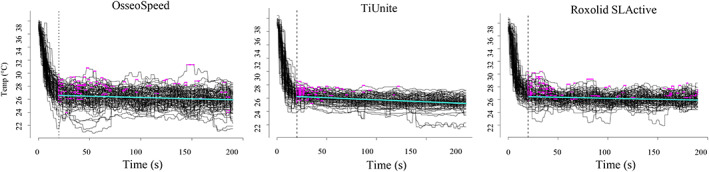
Surface temperature for OsseoSpeed, TiUnite and Roxolid SLActive dental implants during 50 double wavelength laser irradiations recorded using two thermocouples (2 × 50 lines) with max temperatures marked (magenta dots) for each irradiation and mean temperature (horizontal turquoise/light line). Over the first 20 s, initial temperature, 36.0–38.2°C, dropped 8–12°C for all implants (dashed vertical line)

**FIGURE 6:**
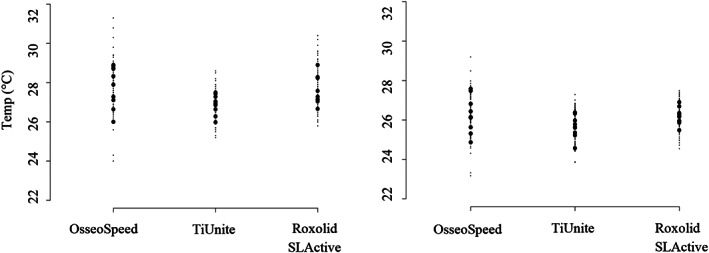
Dot‐plot showing mean of mean maxi and mean temperature for each implant (big dots) and overall variation (small dots)

After 20 s, mean temperatures continued to decrease for all implant systems. The decrease was significantly greater for TiUnite and Roxolid SLActive compared with OsseoSpeed implants (*p* < 0.001). Based on 50 recordings for each implant system, mean max temperature for OsseoSpeed was 27.9°C (SD = 1.3), TiUnite 27.0°C (SD = 0.7), and Roxolid SLActive 27.8°C (SD = 1.0) (Figure 6). There was a variation in the calculated ICCs for the implant systems (0.25–0.70) (Table 3, Figure 5).

**TABLE 3:** Statistical analysis of mean max, mean temperatures, confidence interval and ICC values for five irradiations on 10 implants for each system, measured in the 20–190 s interval of double wavelength irradiation

	OsseoSpeed		TiUnite		Roxolid SLActive	
	Mean max temp	Mean temp	Mean max temp	Mean temp	Mean max temp	Mean temp
1	27.9	26.4	27.3	26.3	28.3	26.9
2	27.1	25.6	27.3	26.3	28.9	26.7
3	26.7	25.3	27.5	26.4	27.6	26.2
4	26.0	24.9	26.9	26.0	28.2	26.3
5	28.7	26.2	26.0	24.6	28.3	26.4
6	28.3	26.2	26.3	25.2	27.1	25.9
7	28.9	26.8	27.1	25.6	27.3	26.2
8	28.8	27.6	27.0	25.8	27.2	26.0
9	28.7	27.5	26.7	25.6	26.7	26.0
10	27.3	26.1	26.9	25.4	27.1	26.0
Mean CI	27.9 (27.6;28.2)	26.3 (26.1;26.5)	27.0 (26.6;27.3)	25.7 (25.5;26.0)	27.8 (27.4;27.9)	26.2 (26.0;26.4)
ICC	0.61	0.63	0.46	0.70	0.38	0.25

*Note*: Temperature in °C, CI = 95% confidence interval (W) based on 50 irradiations/ implant system, ICC = Intraclass correlation coefficient based on five irradiations on 10 implants for each implant system.

Differences in mean max temperature and mean temperature between OsseoSpeed and TiUnite implants were statistically significant (*p* < 0.001). Based on 51.250 recordings (at 1‐s intervals) from the two thermo‐couplers, there was a probability of less than 0.1% to reach a temperature greater or equal to 28.7°C for TiUnite, 29.2°C for Roxolid SLActive, and 31.5°C for OsseoSpeed implants. Quality control of the fiber tip revealed minor damages for 5 out of 150 irradiations not affecting the power output. For the post hoc sample size calculation, using the observed study parameters (ma = 28.50, SD = 1.24), a significance level of 1.0% (α = 0.010) and a power of 99.0% (1‐β = 0.990), we calculated that three (3) implants would be sufficient to provide statistical relevant temperature differences compared with 47°C.

## 4 DISCUSSION

The main aim of this study was to investigate whether calibrated double wavelength laser irradiation using 2780 nm Er,Cr:YSGG and 940 nm diode lasers might critically influence the implant surface temperature. The results did not demonstrate an increase in surface temperature from the 37°C at baseline, indicating that calibrated double wavelength laser irradiation safely can be used in vitro of titanium dental implants. However, after an initial phase of temperature drop, the temperature slowly decreased over time and significant temperature differences were observed among the implant systems. Therefore, the null hypothesis, that the final temperature would be similar for the different implant systems, was rejected. Based on observed study parameters, we found that a sample size less than the number of implants included in the present study would be sufficient to obtain an adequate study power.

### 4.1 Calibration

The laser devices use optical fiber for light energy transmission with losses of light energy. In the calibration part, mean measured output power compared with device setting was approximately 20% lower for both Er,Cr:YSGG and 940 nm diode lasers. This is in accordance with manufacturer information and recent studies (Al‐Karadaghi et al., 2015; Gutknecht et al., 2016). Nevertheless, we found differences related to power settings. At low power the differences were only 10% and at high power up to 24%, indicating that every output power/energy needs to be controlled and calibrated for each irradiation.

For the calibration of the Er,Cr:YSGG laser, only small differences were observed in initial temperature at different power settings following a 30‐s irradiation. An inside implant temperature variation between 22 and 25°C was recorded. The findings are congruent to those in an in vitro study where a temperature of 20°C was measured following Er,Cr:YSGG laser irradiation on SLA titanium discs at a power setting of 0.5, 1.0 and 1.5 W and a distance of 0.5 mm when water/air‐spray was activated (Strever et al., 2017).

With water/air‐spray, 940 nm diode laser irradiation with different power settings only slightly influenced the surface temperature. In contrast, without water cooling, a rapid increase in temperature was observed following increased output energy for all implant systems. OsseoSpeed and TiUnite reached a temperature 45°C higher than Roxolid SLActive at output power of 3.3 W (power setting 4.25 W). The laser energy absorption coefficient of the surface and the thermal conductivity of the core titanium material differ between the implant systems. Core material of OsseoSpeed and TiU are pure Grade IV titanium, whereas Roxolid SLActive is a titanium‐zirconium alloy with a lower thermal conductivity. These findings confirm previous studies showing that the temperature is affected by the laser wavelength (Valente et al., 2017; Leja et al., 2013; Geminiani et al., 2012) and titanium surface composition (Giannelli et al., 2015; Matys et al., 2016). These studies also report a positive association between increased power/energy density and increased temperature without water‐cooling, underlining the importance of water‐cooling during irradiation of Er:YAG, Er,Cr:YSGG and /or diode lasers on titanium surfaces.

The highest Er,Cr:YSGG laser output power not causing micro‐texture alterations was 1.8 W for the Roxolid SLActive, 0.9 W for TiUnite, and 1.1 W for the OsseoSpeed surface. These findings indicate an implant system specific interaction between output laser energy and implant surface composition. In still another study, evaluating the effect of different laser wavelength on titanium discs, similar interaction between wavelength, surface alteration, surface chemistry and output energy was observed (Park et al., 2012).

To study thermal and decontamination effects of Er,Cr:YSGG (Gholami et al., 2018; Takagi et al., 2018) or Er:YAG lasers (Al‐Hashedi et al., 2017; Larsen et al., 2017; Matys et al., 2016) on titanium surfaces, a distance of 0.5–1.5 mm between fiber tip end and implant surface has been used. Diode laser has been tested at a distance of 2–5 mm (Geminiani et al., 2012; Leja et al., 2013; Valente et al., 2017). These differences may greatly impact outcomes. In one study, robotic guidance used a custom computer‐controlled program to regulate the movement in a bidirectional raster scan pattern for handpiece positioning and movement at a 0.5 mm distance (Strever et al., 2017). The present study used a computer numerical control (CNC) device with ability to program movement of the laser handpiece and implant in a reproducible pattern. By mounting the laser handpiece on a sliding board, the distance was adjusted in the range of 0.995–1.005 mm. The laboratory setup was intended to replicate a clinical setting of free access to the implant surface, potentially irradiating different implant systems perpendicular to the surface (Hauser‐Gerspach et al., 2010; Park et al., 2012; Ercan et al., 2014; Larsen et al., 2017) and at the same time controlling the distance between the fiber tip and the implant.

### 4.2 Experiment

In the experimental part, following 190 s of irradiation, mean temperature for each of the three implant systems decreased to approximately 10°C below the 37°C baseline. Initially, surface temperature dropped consistently, flattening out after 20 s to slightly above the water/air‐spray temperature. Recent in vitro studies demonstrate comparable temperature endpoints following Er,Cr:YSGG laser irradiation of titanium surfaces (Romanos et al., 2017; Strever et al., 2017). Even though power/energy output was not reported, principal findings are congruent with the present study. Addressing the efficacy of debridement and disinfection of titanium surfaces described in recent studies on Er,Cr:YSGG (Ercan et al., 2014; Takagi et al., 2018) and diode lasers (Bach et al., 2000; Deppe & Horch, 2007), the present findings have clinical relevance where longer duration irradiation of contaminated implants might be needed.

After 20 s minor temperature differences were observed among the implant systems. Differences in absorption coefficients and reflectivity or the use of different output powers/energies can probably explain these observations. Similar variations have been shown in other laboratory studies comparing thermal conductivity of different titanium alloys following Er:YAG and diode laser irradiation (Matys et al., 2016) and Er,Cr:YSGG laser irradiation (Gomez‐Santos et al., 2010) on different implant surfaces. Other possibly influencing factors are differences in temperature of the water/air‐spray (range 22.7–23.7°C) and isolation and fixation of the thermo‐couplers. The transmission through the fiber tip may decrease due to damages caused by disrupted particles from the titanium surface, and consequently, reduction of output energy may occur (Taniguchi et al., 2013). However, in the present study, evaluation of the fiber tip revealed only few cases with minor damage not affecting the output energy.

The authors acknowledge several limitations of the study. First, the calibration revealed that specific implant system breakdown thresholds and applied laser energies are not immediately applicable for other lasers or implant systems. Second, only three titanium dental implant surfaces out of a plethora of commercially available surface modifications were examined limiting the general applicability of the observations. Included implants were selected based on frequent use worldwide and representing different surface modifications. Third, a small sample size was applied during the calibration and the evaluation not qualitatively validated, the empirical value of the calibration is limited. In contrary, the sample size for the experimental study was over‐sized compared with the post‐hoc sample size calculation. Moreover, surface micro‐texture evaluation by SEM depicts only 2‐d surface characteristics (Wennerberg & Albrektsson, 2009). However, in the context of no available literature on double wavelength laser energy irradiation on titanium implant surfaces, the information from the calibration was critical for the experimental part of the study. Finally, a repeatable movement pattern and constant distance between the fiber tip end and the implant surface were ensured by the robotic laboratory setup. These standardized in vitro setups are not obtainable in a clinical setting, given the design of today's commercially available fiber tips.

Within the limitations of the study, the following observations can be summarized: Calibrated double wavelength laser irradiation of 2780 nm Er,Cr:YSGG and 940 nm diode lasers did not, under in vitro conditions, critically influence the implant surface temperature for included principal implant systems. During laser irradiation, the temperature decreased rapidly from 37°C to steady‐state levels, of 25.7–26.2°C, close to the water/air‐spray temperature.

Further in vitro and preclinical studies need to be undertaken before double wavelength laser irradiation is deemed reliable to be tested in clinical settings. A laboratory 3‐d implant surface evaluation of the debridement and decontamination efficacy, as well as the biocompatibility of contaminated implants following the double wavelength laser irradiation, are warranted. In addition, improved fiber tip design, ensuring an optimized distance between the tip end and implant surface under sufficient water cooling, is also motivated.

## AUTHOR CONTRIBUTIONS

Authors Peter Fahlstedt, Knut N Leknes conceived the ideas; Peter Fahlstedt collected the data; Stein Atle Lie analyzed the data; and Peter Fahlstedt, Knut N Leknes and Dagmar F Bunæs prepared the manuscript.

## Data Availability

The data that support the findings of this study are available from the corresponding author upon reasonable request.
